# Identifying key parameters for reliable assessment of entomopathogenic nematodes viability as affected by spray application stress‐related factors

**DOI:** 10.1002/ps.8847

**Published:** 2025-04-26

**Authors:** Roberto Beltrán‐Martí, Marco Resecco, Elena Gonella, Sofía Victoria Prieto, Marco Pittarello, Cruz Garcerá, Patricia Chueca, Alberto Alma, Fabrizio Gioelli, Marco Grella

**Affiliations:** ^1^ Centro de Agroingeniería Instituto Valenciano de Investigaciones Agrarias (IVIA) Valencia Spain; ^2^ Department of Agricultural, Forest and Food Sciences (DiSAFA) University of Turin (UNITO) Grugliasco Italy; ^3^ Department of Veterinary Science (DSV) University of Turin (UNITO) Grugliasco Italy

**Keywords:** biopesticides, biocontrol agents, EPN activity, non‐lethal stress, spray technology, survival

## Abstract

**BACKGROUND:**

Conventional pesticide application equipment (PAE) is used to apply entomopathogenic nematode (EPN)‐based bioinsecticides, but their closed hydraulic systems could raise the temperature of the spray mixture up to 40 °C, potentially harming EPN, since temperatures above 30 °C can immobilize nematodes, reducing their infective capacity. This study aimed to identify the most suitable method to evaluate EPN viability under the effects of PAE technology.

**RESULTS:**

Three EPN species—*Heterorhabditis bacteriophora, Steinernema feltiae,* and *Steinernema carpocapsae*—were exposed to thermal stress (10, 20, 30, and 40 °C for 270 min) to simulate spray application conditions. Three viability evaluation methods were compared: prodding stimulation, NaCl chemical stimulation, and no stimulation. Viability was measured by two parameters depending on the assessment method: % actively EPN moving (activity), or % total live EPN, both actively moving and immobile (survival). Additionally, a novel parameter estimating non‐lethal stress (Δ_nl s_) was defined by measuring the live but inactive EPNs. NaCl stimulation was optimized comparing different concentrations and durations and then set at 0.1 g mL^−1^ for 1 min. Temperature significantly affected EPN viability over time. Temperatures around 20 °C preserved optimal conditions, and above 30 °C negatively affected EPN viability, with mortality close to 80% within 90 min at 40 °C. Prodding (measuring survival) yielded higher viability compared to NaCl and no stimulation, which measured activity. Non‐lethal stress parameter increased accordingly to stress increment showing potential as EPN stress‐marker.

**CONCLUSION:**

The study concluded that combined measurement of survival, activity and non‐lethal stress should be considered in EPN viability assessments when designing PAE to ensure high efficacy of biocontrol agents. © 2025 The Author(s). *Pest Management Science* published by John Wiley & Sons Ltd on behalf of Society of Chemical Industry.

## Graphical abstract

Commercial entomopathogenic nematodes (EPNs) were exposed to stress simulating spray application conditions, for viability assessment using different methods. Temperature impacted non‐lethal stress earlier than survival and activity, emphasizing the importance of measuring these parameters to assess pesticide application equipment (PAE) impact on EPN biocontrol.

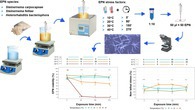

## 1 INTRODUCTION

Entomopathogenic nematodes (EPN) have become a promising and eco‐friendly solution for pest management in agriculture.^1^, ^2^ EPNs naturally parasitize a wide range of insect pests and can therefore act as biological control agents, offering an effective alternative to chemical‐based insecticides for protecting a wide range of crops.^3^, ^4^ Common EPN species, available in commercial product formulations, include *Steinernema carpocapsae* (Weiser, 1955) (Rhabditida: Steinernematidae), *Steinernema feltiae* (Filipjev, 1934) (Rhabditida: Steinernematidae), and *Heterorhabditis bacteriophora* (Poinar, 1976) (Rhabditida: Heterorhabditidae). *Steinernema carpocapsae* employs an ambush strategy, staying near the soil surface and using nictation and jumping to catch the passing near insects.^5^ In contrast, *H. bacteriophora* uses a cruise strategy, actively moving through the soil and using chemical cues to find and penetrate insect hosts.^6^ *Steinernema feltiae* combines both ambush and cruise behaviors, allowing it to adapt to various environments.^7^ Consequently, these nematodes target different pests: *H. bacteriophora* is typically used for soil applications, while *Steinernema* species are used for both soil and foliar applications.^6^, ^8^, ^9^ EPNs display varying levels of host specificity, often exhibiting preferences for a category of insect hosts rather than being strictly species‐specific.^10^ The lethality to the insect host primarily stems from the action of symbiotic bacteria associated with the nematodes, namely *Xenorhabdus* spp., hosted by Steinernematidae, and *Photorhabdus* spp., hosted by Heterorhabditidae.^11^ Once inside the insect host, the nematodes release their associated bacteria into its hemocoel, where they rapidly multiply and produce a range of virulence factors, including toxins and enzymes, leading to the host's death.^2^, ^12^ Additionally, these bacteria contribute to the establishment of a hospitable environment for nematode reproduction by suppressing the growth of opportunistic microorganisms within the insect cadaver.^13^ The nematodes act as carriers for these bacteria, and their ability to effectively transport and introduce them into the insect host is crucial for successful pest control.^14^ Therefore, EPN viability is an essential determinant of their efficacy as biological control agents.^15^

Both for foliar and soil applications, EPN‐based products are typically applied dissolved in water, either by spraying them on vegetation or on the ground. The sprayers used for delivering these formulations are generally the same pesticide application equipment (PAE) used for applying chemical insecticides. Nevertheless, there is a key difference between the products: bio‐insecticides are living organisms that must maintain their viability during and after the application process.^16^ Hydraulic PAE uses the pump to exert pressure on the spray mixture, propelling it through the hydraulic circuit and finally through the nozzle outlets breaking the spray mixture into droplets. The sprayer's hydraulic circuit operates as a closed system, where friction within the pipes, filters, and pump causes the temperature of the spray mixture to increase up to 40 °C or more.^17^ This heat buildup primarily results from the pump's recirculating action, which transfers energy to the mixture throughout the application. This process can impose thermal and physical stresses or even mechanical damage to the living organisms in the bio‐insecticide.^16^ In fact, PAE and associated parameters have been found to have a significant effect on the efficacy and viability of EPNs, showing an inverse relationship with pressure for *H. bacteriophora* and *S. carpocasae*,^18^ and with spray mixture temperature for *H. bacteriophora*.^19^ Based on their results, these authors identified the recommended maximum pressure for EPN delivery and proposed using PAE featured by lower capacity pumps for bio‐pesticides application (e.g., diaphragm, roller pumps) to mitigate this detrimental influence. Besides, Fife *et al*.^20^ found that some hydraulic nozzles may cause damage to EPNs, but with a size larger than the organism, common nozzles were found to be acceptable for spray application of EPNs. Accordingly, Hayes *et al*.^21^ obtained that *S. carpocapsae* induced up to 95% insect mortality even after the infective juveniles had passed through the nozzles of a boom sprayer. In this regard, Anifantis *et al*.^22^ evaluated the effect of hydrostatic pressure on the viability of *S. feltiae* and *H. bacteriophora*, subjecting liquid suspensions of nematodes to eight different pressure levels (5–40 bar) for 20 s. After exposure, EPN viability and infectivity were assessed using bioassays with *Galleria mellonella* (Linnaeus, 1758) (Lepidoptera: Pyralidae) larvae. Although a slight increase in mortality was observed (up to 14% at the highest pressure), statistical analysis did not indicate a significant impact on nematode vitality or infectivity. Moreover, *S. feltiae* was found to be more sensitive to pressure than *H. bacteriophora*, showing a steeper increase in mortality as pressure increased. These results highlight the ability of EPNs to tolerate high‐pressure conditions, paving the way for their application using high‐pressure spraying technologies such as low‐drift nozzles.

Related to the performance of these studies, whose aim is to evaluate the influence of spray operating conditions on the viability of EPNs, it is worth mentioning that one of the most widely used methods to assess EPN viability is the prodding stimulation, which is based on estimating the percentage of mortality and after calculating the percentage of survival.^23^ Straight not‐curved individuals are directly considered dead, while curved non‐motile ones are prodded to check if they are alive or dead. If a prodded non‐motile nematode moves, it is considered alive; if it does not move or breaks, it is considered dead. An alternative method is the chemical stimulation using sodium chloride (NaCl). NaCl stimulation allowed homogeneous viability measurements, producing lower variability than other stimulants, such as acetic acid.^24^ Brusselman *et al*.^25^ used this method to assess the effects of several passages through a centrifugal pump on *S. carpocapsae* viability, evaluating the survival and also the activity, measured as the percentage of actively moving EPNs. These authors observed significant divergence between these parameters, as they recorded a slight reduction of survival after 10 passages, while activity underwent a dramatic drop. Brusselman *et al*.^24^ developed a *S. carpocapsae* viability evaluation method based on NaCl chemical stimulation coupled with an image processing technique to assess the activity, comparing it with prodding stimulation coupled with microscope observation. They recorded a discrepancy between these two methods, with lower values of viability with the NaCl‐image processing technique, since it measured just the activity, but which were more closely aligned with the infectivity of an insect host.

The previously reported divergence in EPN viability depending on the evaluation method^24^, ^25^ highlights the need for a comprehensive assessment of the key parameters that could be useful to assess EPN stress. Accurate estimation of both survival and activity is crucial to determining the true potential of EPN‐based products in pest management practices. For these reasons, the general aim of this study was to identify the key parameters for a reliable assessment of EPN viability as affected by spray application stress‐related factors and the appropriate method to measure them. To achieve this, the following specific objectives were defined: (i) to determine the most appropriate procedure for NaCl stimulation to evaluate EPN activity, (ii) to compare EPN viability measures under laboratory conditions expressed as activity and survival percentages, and (iii) to determine which parameters and which measuring method are most suitable for assessing the effect of application technology stress on EPN viability.

## 2 MATERIALS AND METHODS

### 2.1 EPN commercial formulations

The tests were carried out using commercial formulations containing three of the most widely used EPNs, namely *H. bacteriophora* strain HB1 (Nemagreen®, Biogard, CBC Europe S.r.l., Italy), *S. feltiae* strain SF1 (Nemaplus®, Biogard, CBC Europe S.r.l., Italy), and *S. carpocapsae* strain SC1 (Nemastar®, Biogard, CBC Europe S.r.l., Italy). Nemagreen® is recommended for soil applications to control soil‐dwelling insects (e.g., scarab grubs), while Nemaplus® and Nemastar® can be used for both soil and foliar applications to control a broad range of insect pests (e.g., fruit moths, thrips, beetles).

### 2.2 Experimental design

The viability assessment method was studied as a qualitative factor with three variants: prodding stimulation, chemical stimulation with NaCl, and no stimulation (Table 1). Measurements were conducted after submitting individuals of the three EPN species to both thermal stress and sequential exposure times, simulating in a laboratory‐controlled environment some of the possible conditions that the spray mixture may be subjected to during spray application. To assess the effect of temperature and exposure time to thermal stress, two quantitative factors were analyzed: temperature with four levels (10, 20, 30, and 40 °C), and exposure time at each temperature at four levels (0 [control], 90, 180, and 270 min). The response variable was EPN viability, measured by two parameters depending on the assessment method: (i) prodding stimulation method led to the total percentage of live specimens defined as nematodes survival (%) and (ii) the chemical stimulation and no stimulation methods led to the percentage of specimen actively moving defined as nematodes activity (%) (Table 1).

**Table 1:** Assessment methods used to quantify the entomopathogenic nematode (EPN) viability

Assessment method	Description	Individual cases	EPN viability parameter
Prodding stimulation	Prodding the non‐motile nematodes from the analyzed sample and then classifying individuals according to cases through microscope visual analysis	Live nematode: (i) motile + (ii) non‐motile but responsive	Nematodes survival (%)
		Dead nematode: (i) non‐motile and unresponsive + (ii) damaged + (iii) broken in parts	
Chemical stimulation	Adding NaCl to the analyzed sample and then classifying individuals according to cases through microscope visual analysis	Active nematode: (i) motile	Nematodes activity (%)
		Inactive nematode: (i) non‐motile + (ii) damaged + (iii) broken in parts	
No stimulation	Classifying individuals according to cases through microscope visual analysis	Active nematode: (i) motile	Nematodes activity (%)
		Inactive nematode: (i) non‐motile + (ii) damaged + (iii) broken in parts	

For the chemical stimulation method, the addition of a NaCl solution, known as effective nematode motility stimulant, was foreseen^24^ (Table 1). To this extent, preliminary tests were carried out to determine optimal NaCl concentration and the exposure time to NaCl to be used for the evaluation of EPN viability. The optimal combination of NaCl concentration and exposure time was selected to maximize the detection of active individuals, thereby reducing bias from alive but non‐motile nematodes and improving data reliability. In these tests, the quantitative factors were NaCl concentration at two levels (0.1 and 0.2 g mL^−1^) and exposure time to NaCl at four levels (1, 3, 5, and 7 min). The qualitative factor was the species, with three variants: *H. bacteriophora*, *S. feltiae*, and *S. carpocapsae*.

The NaCl concentration of 0.1 g mL^−1^ and an exposure time of 1 min were chosen based on preliminary tests, which indicated that this combination optimized EPN activity while reducing the risk of over‐stress. Specifically, 0.1 g mL^−1^ NaCl resulted in higher activity compared to 0.2 g mL^−1^ at 20 °C, with no significant differences observed at other temperatures.

### 2.3 Evaluation of thermal stress and exposure time effect on EPN‐viability

For each tested temperature, three solutions were prepared in a Pyrex beaker using 500 mL of deionized water and 2.85 g of the respective EPN commercial formulation (corresponding to 6.25 × 10^6^ IJs/L), based on the label concentration. To prepare the solutions at 20, 30 and 40 °C, water was first heated to reach the desired temperature using an Arex 6 heating magnetic stirrer (VELP Scientifica, Italy). After reaching the temperature, the formulation was added and mixed in the water suspension using the stirrer for 1 min until fully homogenized. Once homogenized, the solutions were incubated into a Julabo TW8 water bath (Julabo GmbH, Germany) to maintain a constant temperature throughout the test duration (270 min). For the solutions at 10 °C, the water was cooled to 10 °C and incubated in a FOC 200I cooled incubator (VELP Scientifica, Italy). Ice packs were used as necessary to maintain a constant temperature throughout the trial duration when the beakers were out of the incubator for sampling. In all cases, the temperature was controlled using thermocouples, one per beaker, submerged in the solution and connected to a HD32.8.16 datalogger (Delta OHM S.r.l., Italy) acquiring data at 0.5 Hz.

From each solution, 1 mL was sampled at four defined times, 0, 90, 180, and 270 min. The control sample was taken right after the suspension had been homogenized. Each sample was diluted to 1:10 in deionized water. Then three sub‐samples of 50 μL were pipetted on a plain microscope slide and observed by using an SZH‐10 stereo microscope (Olympus, Japan) to evaluate viability with the corresponding assessment method. Three replicates for each temperature, EPN species, time exposure and assessment method were carried out. Concerning the preliminary tests aimed to determine the optimal NaCl concentration and the exposure time to NaCl, also in this case three replicates for each temperature, EPN, time exposure, NaCl concentration and exposure time to NaCl were carried out.

### 2.4 EPN viability parameters calculation

The nematodes viability was estimated according to the applied method. Percentage of nematodes survival (%) was used for prodding stimulation method, whereas percentage of nematodes activity (%) was used for NaCl chemical stimulation and no stimulation methods (Table 1).

Independently from the method used, once the sub‐sample was ready for evaluation, the first step consisted of counting the total number of individuals. Then the individuals in the sub‐sample were classified and counted according to the measured features for each assessment method (Table 1). Finally, the percentage of live/active and dead/inactive nematodes was determined according to the methods.

For prodding stimulation, straight not‐curved individuals were considered dead, while curved immobile nematodes were prodded by using a needle. If the nematodes were already actively moving or reacted to the mechanical stimulation (individual movement detected after prodding), they were considered alive. If they did not move when prodded or were damaged or broken in parts, they were considered dead. Based on the total nematodes and the dead nematodes counted in the sub‐sample, the nematode survival expressed in % was calculated according to the Eqn (1):

(1) $$\mathrm{NS}=\frac{\mathrm{TN}-\mathrm{DN}}{\mathrm{TN}}\times 100$$

where NS is nematode survival (%); TN is the total number of nematodes (n.°); DN is the number of dead nematodes (n.°).

For chemical stimulation, NaCl was used to stimulate the EPN movement in the solution. A volume of 25 μL of the respective NaCl concentration (0.1 or 0.2 g mL^−1^) was directly added to the EPN suspension already pipetted on the glass slide. Individuals that were actively moving were then counted after 1, 3, 5, and 7 min of exposure to NaCl. In this case, if nematodes did not move or were damaged or broken in parts, they were considered inactive.

Similarly, for the no stimulation method the number of actively moving individuals in the undisturbed subsample was determined by directly counting the number of moving nematodes. Also in this case, if nematodes did not move or were damaged or broken in parts, they were considered inactive.

Therefore, for the (i) chemical stimulation and (ii) no stimulation methods, based on the total nematodes and the moving nematodes counted in the sub‐sample, the nematode activity expressed in % was calculated according to the Eqn (2):

(2) $$\mathrm{NA}=\frac{A}{\mathrm{TN}}\times 100$$

where NA is nematode activity (%); TN is the number of total nematodes (n.°); A is the number of active nematodes (n.°).

A third parameter was calculated to describe the EPN stress level associated with the tested stressor, by estimating the level of divergence between the chemical stimulation and no‐stimulation methods respect the prodding stimulation method, that is the percentage of live but inactive nematodes, corresponding to non‐lethal stress. In principle, the higher the EPN stress level, the lower the EPN activity before leading individuals to death. Therefore, the Δ_nl s_ parameter expressed as % was calculated according to the Eqn (3):

(3) $${∆}_{\mathrm{nl}\;s}=\mathrm{NS}-\mathrm{NA}$$

where Δ_nl s_ is a percentage of live but inactive nematodes (%); NS is the percentage of nematode survival derived from prodding stimulation method; NA is the percentage of nematode activity obtained from the corresponding chemical stimulation or no stimulation methods.

### 2.5 Data management and statistical analysis

Data were analyzed using a series of Generalized Linear Mixed Models (GLMMs) in the R environment,^26^ each one for different purposes.

The first analysis was aimed at identifying the optimal NaCl concentration (0.1 and 0.2 g mL^−1^) and exposure time to NaCl (1, 3, 5 and 7 min) for the NA (%) measurements based on chemical stimulation method. Initially, the relationship between NA (%) and exposure time to NaCl was described for the two NaCl concentrations, separately for each EPN species and temperature. For this purpose, the mean and standard error of NA (%) were calculated for the aforementioned combinations and represented graphically (Fig. S1). Based on these descriptive analyses, which indicated an effect of temperature rather than EPN species or exposure time to NaCl, the models were constructed separately for each temperature (10, 20, 30, and 40 °C). In each model, the NA (%) was set as the response variable, whereas the NaCl concentration, exposure time to NaCl, and their interaction included as fixed factors. The sampling tube, nested within nematode species (i.e., *H. bacteriophora*, *S. carpocapse* and *S. feltiae*), was treated as a random factor to account for the potential variability introduced by the sampling process for each species. Additionally, exposure time (0, 90, 180, and 270 min) was included as a random factor to account for repeated measurements of each tube over time. From this point on, only the data retrieved from the optimal NaCl stimulation procedure, as the best combination of NaCl concentration and time exposure to NaCl, were considered for the subsequent statistical analysis.

To assess if the three assessment methods (i.e., prodding stimulation, NaCl chemical stimulation and no stimulation) had an overall effect on EPN viability, a GLMM was carried out. Viability (i.e., NS% and NA%) was set as the response variable and the assessment method as a fixed factor. The sampling tube, nested within the nematode species, was treated as a random factor to account for the potential variability introduced by the sampling process for each species. Additionally, exposure time was included as a random factor to account for repeated measurements of each tube over time. Since the objective was not to evaluate the effect of temperature in this case, this factor was not specified as either a fixed or random factor to maintain focus on the general effects of the assessment methods.

Once defined the overall effect of assessment method on the EPN viability measurement, the effects of temperature and exposure time on EPN viability were investigated accounting for the variability introduced by the EPN species and by the assessment methods. Firstly, the mean and standard error of EPN viability were calculated for each combination of temperature and exposure time, respectively for each EPN species and assessment method. These means were represented graphically (Fig. S2) to visually describe the relationship between temperature and exposure time. Based on these descriptive analyses, a GLMM was used to investigate the effects of temperature and exposure time on EPN viability, considering the variability associated with the three EPN species and to the assessment method. Therefore, EPN viability was used as the response variable, while temperature, exposure time, and their interaction were included as fixed factors. To take into account the repeated measure structure and the variability associated with the temperature and EPN species, a random factor with assessment method nested within the sampling tube, which was further nested within nematode species was specified in the model.

The final analysis aimed to evaluate the effect of exposure time and temperature on the ∆_nl s_, accounting for the variability introduced by the EPN species and by the two assessment methods: NaCl chemical stimulation and no stimulation (Section 2.4). For this purpose, the mean and standard error of ∆_nl s_ were preliminarily calculated for the combinations of exposure time and temperature for each assessment method, respectively for each EPN species. Based on the graphical representation of these values (Fig. S2), a GLMM was performed, specifying Δnl s as the dependent variable and temperature, exposure time, and their interaction as fixed factors. To account for the repeated measure's structure and the variability associated with temperature and EPN species, a random factor was defined with the assessment method nested within the sampling tube, which in turn was nested within nematode species. Noteworthy, in agreement with the principle that the higher the EPN stress level the lower will be the EPN activity before leading individual to death, in this last GLMM analysis the EPN viability equal to 0% was excluded from the dataset. The ∆_nl s_ parameter, as conceived, can be used to describe the non‐lethal EPN stress associated with the tested stressor. Therefore, EPN viability reaching values equal to 0% is actually describing the death of all individuals instead of EPN stress level.

In all models, the response variable, being a percentage, was modeled using the beta distribution, suitable for variables constrained within the 0–1 interval. Since the beta distribution does not accept exact 0 or 1 values, percentage values were pre‐processed using the Smithson and Verkuilen^27^ transformation.

The models were implemented using the ‘glmmTMB’ function of the glmmTMB package.^28^ The significance of fixed and interaction effects was tested using the ‘joint_tests’ function from the EMMEANS package.^29^ For significant fixed or interaction effects, Tukey's post‐hoc tests were performed using the emmeans function from the EMMEANS package. The significance threshold was set at *P* < 0.05.

## 3 RESULTS

### 3.1 NaCl concentration and exposure time chemical stimulation effects

**Figure 1:**
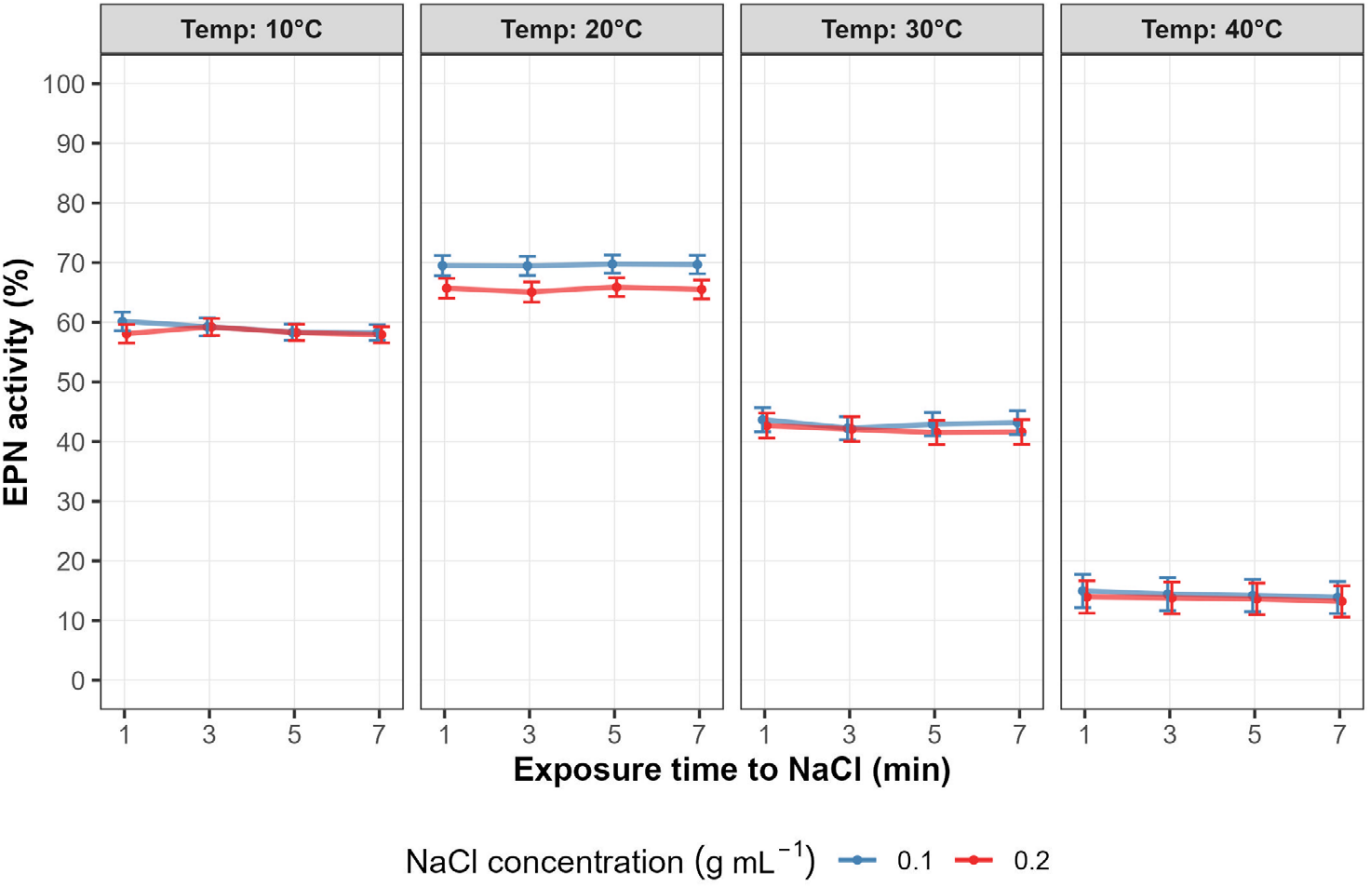
Relationship between EPN activity (%) and NaCl exposure time (1, 3, 5, and 7 min) for the two NaCl concentrations (0.1 and 0.2 g mL^−1^) by using chemical stimulation. Plots are split by temperatures (10, 20, 30, and 40 °C) used as thermal stress to EPN. Average values ± standard error of the mean (SEM) is represented.

The results of GLMM analysis are shown in Table 2. Neither the main effects of NaCl concentration and NaCl exposure time, nor their interaction were statistically significant (*P* > 0.05) on EPN activity at 10, 30, and 40 °C. Meanwhile, at 20 °C a significant effect of NaCl concentration on EPN activity was detected (*P* < 0.001). At 20 °C the average individual's activity for the concentration of 0.1 g mL^−1^ was 69.60% ± 0.79 standard error of the mean (SEM), while using a concentration of 0.2 g mL^−1^ resulted in a significant lower activity, averaging equal to 65.55% ± 0.81 SEM (Fig. 1). Concurrently, at 20 °C there was no significant effect of NaCl exposure time and its interaction with NaCl concentration. Additionally, the EPN response when subjected to NaCl chemical stimulation (A) and exposure time (B) showed similar trend for the three EPN species (*H. bacteriophora*, *S. carpocapse* and *S. feltiae*) tested (Fig. S1). In all cases the average values for the nematode activity were higher or at least equal when 0.1 g mL^−1^ NaCl was added compared to 0.2 g mL^−1^ NaCl (Fig. 1). Based on these findings, the chemical stimulation method using the lowest stress, i.e., 0.1 g mL^−1^ NaCl concentration with a 1‐min exposure time, was selected for the subsequent trials.

**Table 2:** Generalized linear mixed models (GLMMs) results (*P* < 0.05) for the nematode activity (%) measured by applying the sodium chloride (NaCl) chemical stimulation method

	Nematode activity (%)				
Model terms	DF1	F. ratio	*χ* ^2^	*P*‐value	Signif.^†^
10 °C					
Main effects					
NaCl concentration (A)	1	0.829	3.306	0.363	NS
NaCl exposure time (B)	3	1.102	0.829	0.347	NS
Interactions					
A × B	3	0.437	1.311	0.727	NS
20 °C					
Main effects					
NaCl concentration (A)	1	23.678	1.143	< 0.001	***
NaCl exposure time (B)	3	0.381	23.628	0.767	NS
Interactions					
A × B	3	0.196	0.588	0.900	NS
30 °C					
Main effects					
NaCl concentration (A)	1	0.996	2.898	0.408	NS
NaCl exposure time (B)	3	3.305	3.305	0.069	NS
Interactions					
A × B	3	0.285	0.855	0.836	NS
40 °C					
Main effects					
NaCl concentration (A)	1	0.394	1.182	0.757	NS
NaCl exposure time (B)	3	1.678	1.687	0.194	NS
Interactions					
A × B	3	0.049	0.147	0.986	NS

^†^ Significance levels: NS: *P* > 0.05, *: *P* < 0.05, **: *P* < 0.01, ***: *P* < 0.001.

### 3.2 EPN viability as affected by laboratory assessment method used

**Figure 2:**
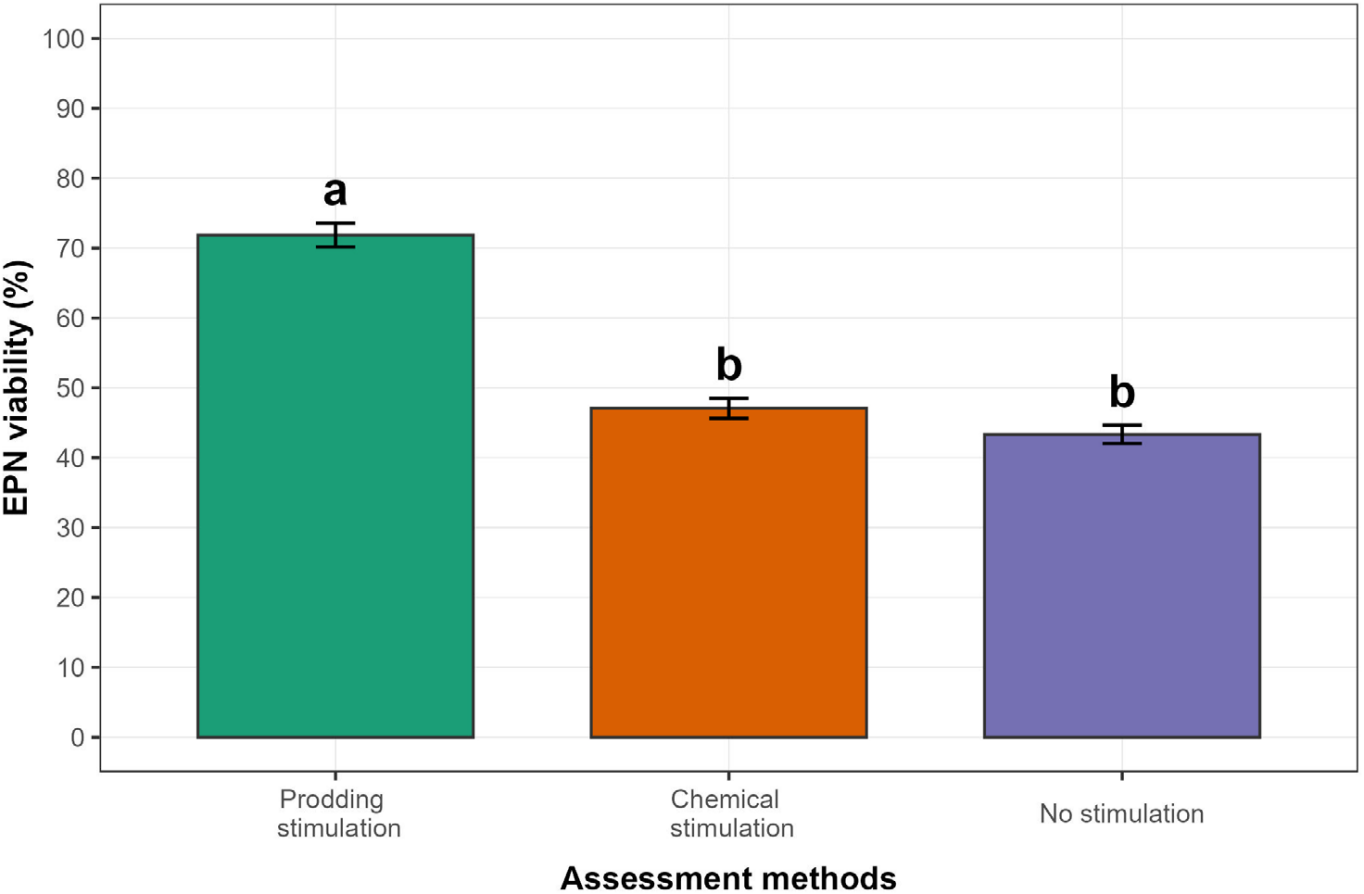
Entomopathogenic nematode (EPN) viability (%) described by nematode survival (NS %) and nematode activity (NA %) parameters according to measurement method used. Different letters denote significant differences defined according to Tukey's *post hoc* tests (*P* < 0.05). Bars represent average values ± standard error of the mean (SEM).

GLMM analysis confirmed that the EPN viability is significantly affected by the laboratory assessment method adopted for its measurements [*F*(2, ∞) = 147.575, *χ* ^2^ = 295.150, *P* < 0.0001]. Prodding method achieved significantly higher values, equal to 71.87% ± 1.71 SEM, compared to chemical stimulation (NaCl at 0.1 g mL^−1^ concentration and 1 min NaCl exposure defined based the output described in Section 3.1) and no stimulation methods, which reached average values of 47.07% ± 1.44 SEM and 43.35% ± 1.31 SEM, respectively (Fig. 2). Noteworthy, NaCl stimulation and no stimulation did not significantly differ (Fig. 2). These results confirmed that the average EPN viability assessed through prodding (i.e., viability = survival) is at least 25% higher than those assessed applying any of the other two methods, where viability is considered the activity.

### 3.3 Temperature and time exposure effect on EPN viability

A similar trend of EPN viability was recorded in relation to the exposure time and temperature for all nematode species and for the three methods used (Fig. S2). For this reason, a single GLMM was used, grouping survival and activity data (i.e., results obtained using prodding, chemical stimulation and no stimulation) from the three EPNs (Section 2.5).

**Figure 3:**
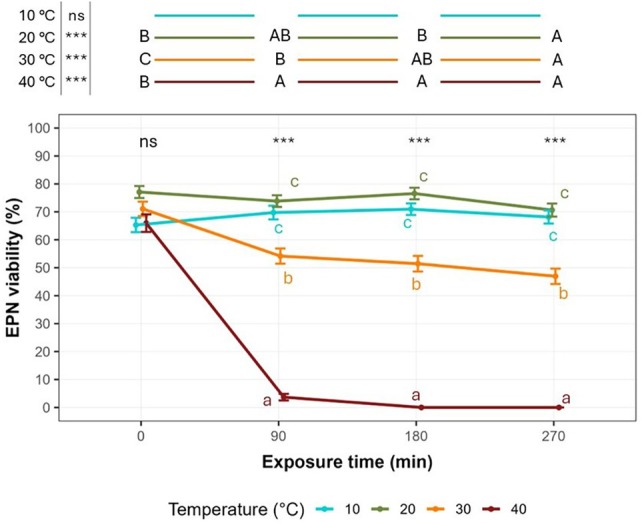
Relationship between entomopathogenic nematode (EPN) viability (%) and temperature exposure time for the temperatures used for thermal stress of EPNs. Different lowercase letters indicate significant differences between the temperatures within each exposure time, whereas uppercase letters indicate significant differences between the exposure times within each temperature. The differences are defined according to Tukey's *post hoc* tests (*P* < 0.05). Average values ± standard error of the mean (SEM) are represented.

Figure 3 shows the effect of temperature and exposure time interaction on the EPN viability (%). GLMM results indicated that the interaction between temperature and exposure time significantly affected the EPN viability [*F*(9, ∞) = 239.851, *χ* ^2^ = 2158.659, *P* < 0.0001]. Temperature significantly affected nematode viability over time, excepting at control time (0 min) where EPN viability was not different across temperatures. When EPNs were exposed to 40 °C, viability dropped to 3.69% ± 1.18 SEM at 90 min, reaching 0% at 180 and 270 min. In contrast, 20 °C induced minimal stress on individuals, with no significant differences in viability over time when compared to 10 °C. At 30 °C the viability significantly decreased compared to 10 and 20 °C, by 54.17% ± 2.72 SEM at 180 and 270 min of exposure (Fig. 3). Concerning the effects of exposure time at each temperature, at 10 °C, EPN viability showed no significant differences among any exposure time, with viability percentages remaining relatively stable, being 70.95% ± 2.08 SEM the maximum viability percentage at 180 min. At 20 °C, the EPN viability significantly decreased after 270 min of exposure compared to both control time (0 min–highest average EPN viability equal to 77.10% ± 2.15 SEM) and 180 min; intermediate EPN viability values were detected after 90 min of exposure. At 30 °C, a significant drop of EPN viability from the control time (0 min) to other exposure times (90, 180, and 270 min) was detected, with no significant differences between exposure times of 90, 180, and 270 min. Viability decreased from 71.07% ± 2.59 SEM (0 min) to 54.17% ± 2.72 SEM (90 min), 51.44% ± 2.79 SEM (180 min), and 46.94% ± 2.76 SEM (270 min). At 40 °C, nematodes showed viability comparable to other temperatures at 0 min, whereas already after 90 min EPNs became unviable due to the temperature exposure. In general, the exposure time played a crucial role on EPN viability especially for the higher temperatures (30 and 40 °C). Indeed, at 30 °C the exposure time deeply affected the number of viable nematodes (within 270 min) while at 40 °C the exposure time brought viability close to zero already after a short time (90 min).

### 3.4 EPN stress level quantification through Δ_nl s_ (%) parameter

The Δ_nl s_ (%), indicating the difference between survival and activity (Section 2.4), was calculated to measure the percentage of nematodes that were alive but inactive due to the stress induction, hence representing the non‐lethal EPN stress level associated with the stress factor tested. Considering that, the plots in Fig. S3 display similar trend of Δ_nl s_ (%) in relation to the exposure time (min) for both methods used to calculate activity (i.e., chemical stimulation and no stimulation) within each temperature, those data were grouped in a single GLMM. Additionally, the data obtained at 40 °C temperature was excluded from dataset and not included the statistical analysis (Section 2.5). Indeed, at 40 °C the EPN viability approaches 0% immediately (Fig. 3) so the Δ_nl s_ was 0% (no presence of live but inactive nematodes because all died due to exposure to high temperature) in most cases (Fig. S3).

**Figure 4:**
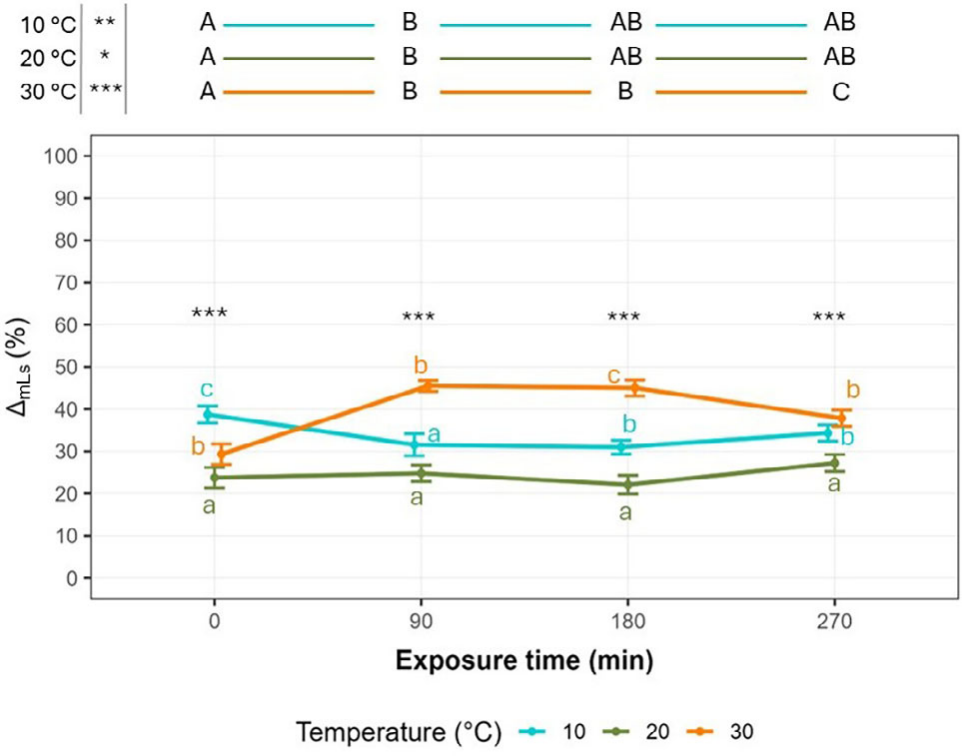
Relationship between Δ_nl s_ (%) and exposure time (0, 90, 180 and 270 min) for the temperatures (10, 20, and 30 °C) used as a thermal stress to EPNs. Different lowercase letters indicate significant differences between the temperatures within each temperature exposure time, whereas uppercase letters indicate significant differences between the temperature exposure time within each temperature. The differences are defined according to Tukey's *post hoc* tests (*P* < 0.05). Average values ± standard error of the mean (SEM) are represented.

In Fig. 4 the effects of temperature and exposure time interaction on the Δ_nl s_ (%) are reported. GLMM results indicated that the interaction between temperature and exposure time significantly affected the Δ_nl s_ [*F*(6, ∞) = 10.920, *χ* ^2^ = 65.520, *P* < 0.0001]. In general, the lowest values were detected at 20 °C (below 30%) remaining consistent across the exposure times. At 10 °C the Δ_nl s_ showed higher values compared to 20 °C ranging between 30 and 40% across the exposure times. According to the results on EPN viability (Fig. 3) the 20 °C temperature determined the lowest thermal stress to EPNs without effects of exposure time. Meanwhile the coolest condition (10 °C) determined higher stress to nematode compared to 20 °C showing Δ_nl s_ values in between of those measured for 20 and 30 °C at 90, 180 and 270 min temperature exposure time. At 30 °C, Δ_nl s_ values peaked around 45–50%, with an initial Δ_nl s_ of 29.15% ± 2.46 SEM at control time (0 min) that rose to a maximum of 45.52% ± 1.32 SEM after 90 min of exposure, indicating a substantial reduction of EPN activity induced by thermal and exposure stress. The temperature significantly affected Δ_nl s_ parameter over time. Differently from the EPN viability (Fig. 3), Δ_nl s_ parameters related to the EPN stress were already significantly different at 0 min (control time) with the lowest values at 20 °C (24.0% ± 2.73), followed by 30 and 10 °C (Fig. 4). At 90 min exposure, significant differences were observed between 30 °C and both 10 and 20 °C; with statistical differences detected between 10 and 20 °C. After 180 min, significant differences between the three temperatures were detected with the highest value Δ_nl s_ measured at 30 °C (43.38% ± 2.35 SEM). After 270 min, Δ_nl s_ values increased slightly for 10 °C (38.44% ± 2.32 SEM) and for 20 °C (32.38% ± 2.97 SEM) meanwhile slightly decreased for 30 °C (34.73% ± 2.83 SEM) with significant differences between 20 °C and both 10 and 30 °C; no significant difference was detected between 10 and 30 °C (Fig. 4). Concerning the effects of exposure time at each temperature, at 10 °C Δ_nl s_ no significant differences were shown among any exposure time, indicating comparable EPN stress mostly induced by temperature itself. At 20 °C, significant higher Δ_nl s_ values compared to other exposure times were measured at 270 min. After 90 min of exposure at 20 °C the Δ_nl s_ reached intermediate values, not significantly different from those obtained at 0 and 180 min, which were the lowest ones, and that obtained at 270 min, which was the highest one. As expected, the 30 °C was the temperature that mostly induced stress to EPNs, especially during the trial's duration. This was confirmed by Δ_nl s_ values that at the control time (0 min) showed the lowest delta value within 30 °C. Δ_nl s_ significantly increased when passing from 0 to 90 min exposure, when it reached the peak, and then at 180 min the Δ_nl s_ value slightly decreased, but it was not significantly different from the one detected at 90 min of exposure. After 270 min the average Δ_nl s_ value strongly reduced compared to that measured at 180 min of exposure even if they were not significantly different because Δ_nl s_ value at 180 min reached intermediate values between those measured for 90 and 270 min.

## 4 DISCUSSION

This study enabled the selection of the most suitable method for evaluating the effect of spray application stress on EPN viability. According to Brusselman *et al*.,^24^ NaCl chemical stimulation method creates hyperosmotic conditions that can induce nematodes movement by triggering an avoidance response. In our study it was observed that when the NaCl chemical stimulation was applied, the effect of NaCl did not vary significantly over exposure time at all temperatures tested. From a practical standpoint, this means that experimental procedures using NaCl stimulation would not require prolonged waiting periods before microscope measurements, as 1 min was sufficient to allow reliable counting. Regarding NaCl concentration, although it influenced the activity at 20 °C, the magnitude of the activity difference between the two NaCl concentrations was only 4%. It could be considered negligible, especially since no effect of NaCl concentration was observed at any of the other tested temperatures. This suggests that lower NaCl concentrations might be preferable, as they strike a favorable balance by promoting movement without over‐stressing the nematodes. Nonetheless, in our study, NaCl stimulation did not show any significant difference in assessing nematode activity compared with no stimulation method, therefore NaCl exposure was not able to significantly activate nematodes that were live but inactive, irrespective of NaCl concentration and time exposure. Therefore, no advantages compared to the no stimulation method were noticed for the NaCl chemical stimulation.

When comparing the measurement methods assessing survival or activity, in agreement with previous reports,^24^ we consistently found a significant difference, with survival (measured by prodding) displaying higher values than activity (either measured by chemical or no stimulation). It derives that for a comprehensive evaluation of EPN viability it would be necessary to measure both nematodes survival and activity (as they significantly diverge), and the most suitable methods are prodding and no stimulation, respectively. The two parameters can be considered complementary for a reliable assessment of EPN viability (%) for an exhaustive evaluation of spray application technologies effect on EPN efficacy at the time of application, considering that sprayers not only kill the nematodes but also make them inactive, thus potentially affecting their infectivity and the final efficacy in pest control. EPN activity is essential for their mobility in the environment and therefore to guarantee their potential effectiveness as BCA, as their ability to locate and infect hosts highly depends on their foraging strategy and movement capabilities to adapt to various environments and pest behaviors. For example, cruising nematodes, such as *Heterorhabditis* spp., actively move through the soil to locate sedentary pests, such as larvae buried in the soil, which do not move significantly.^6^ Even for ambushing nematodes, such as *S. carpocapsae*, that show limited movement accompanied by nictation (i.e., standing upright and waiting for mobile surface‐dwelling pests),^30^ movement capability is important. Indeed, it is also critical for overcoming physical barriers in the soil, such as variations in texture and moisture, which expand their range of action. Without active movement, EPNs would be unable to adapt to the ecological characteristics of their hosts or reach the microhabitats where pests are located, severely limiting their efficacy as BCA. On the other hand, in particular environmental conditions where limited movement is needed to find a host, live but inactive individuals can still contribute to the final EPN efficacy. Our results, by including observations on EPN species that display different behaviors, highlight how activity and survival are affected by stressors irrespective of behavioral traits; specific studies focusing on a single species may provide a finer description of the response in terms of cruising or ambushing capability.

The evaluation of temperature and time exposure stress showed that the effect of each stressor depended on the effect of the other one, as expected. The 20 °C temperature was identified as the optimal one, as it induced a lower stress irrespective of time exposure. It derives that at the optimal temperature the exposure time has no or only a marginal effect on both EPN viability and EPN stress levels even after a long exposure time (270 min). In contrast, lower (10 °C) or higher (30 °C) temperature caused EPN stress to different extents, with higher heat‐induced stress than cool‐induced stress. Moreover, exposure at 10 °C was not lethal for the nematodes, indicating that lower temperatures are only detrimental for activity but not for survival. These findings are supported by results obtained by El Khoury *et al*.^31^; even if these authors did not study the nematodes activity, they reported that Heterorhabditidae and Steinernematidae EPNs at 20 °C are effectively infective meanwhile at 10 °C they are very little infective, suggesting that EPN activity is strictly related to infectivity. Also, Kung *et al*.^32^ determined that 25 °C is the optimal temperature for EPN survival, further supporting our findings.

Also, the exposure time duration played a crucial role on EPN viability, especially when temperatures were higher than 20 °C. At 30 °C after 90 min the viability was significantly reduced compared to the values at the beginning of the measurements, and subsequently the EPN viability remained stable up to 270 min; meanwhile at 40 °C the EPNs were only viable at 0 min. This suggests that during spray application the EPN‐based spray mixture needs to be maintained at temperatures close to 20 °C and lower than 30 °C for a longer time, while they can resist at a temperature close to 30 °C for a short time. Temperatures higher than 30 °C were in all cases detrimental for EPNs, leading to the total nematodes' death in a short time. This is in line with Grella *et al*.,^17^ who exposed microbial biological control agents (BCA) to temperature and exposure stress, finding that at 40 °C the fungus *Trichoderma harzianum* Rifai (Hypocreales: Hypocreaceae) strain T22 immediately died meanwhile the bacterium *Bacillus amyloliquefaciens* strain QST713 resisted for a short time (less than 60 min) before dying. It would be useful for future research to explore the impact of 40 °C on EPNs for up to 90 min, assessing the effects at different time intervals. This would provide more precise information on how long it takes for EPNs to lose activity or die at this temperature.

A major outcome of this study was the identification of a novel parameter to assess the non‐lethal stress level, namely Δ_nl s_. This parameter was significantly affected by the interaction between temperature and exposure time, showing a trend that was partially divergent from the results obtained by grouping survival and activity. In general, the Δ_nl s_ increased according to the stress induced to the EPNs, indicating that as stress levels rose, a greater proportion of individuals were alive but unable to move on their own (inactive) before reaching a critical stress lethal threshold. This results in faster response to the stress of Δ_nl s_ compared to overall viability, considering that non‐lethal stress effects appear before the lethal ones. For example, at 10 °C the overall viability was stable over time, whereas a significant decrement of Δ_nl s_ was recorded after 90 min. The quick increment of non‐lethal stress further highlights the importance of measuring the relation between EPN survival and their activity. EPN‐individuals depend on their ability to move through soil and navigate diverse habitats to locate insect hosts.^5^, ^6^, ^7^ For this reason, the differences between nematode survival (NS %) and nematode activity (NA %) parameters underscore the need for a comprehensive EPN viability assessment that considers not only their survival once applied but also their potential capability to move when delivered into environment (e.g., soil, leaf, etc.). In this study, the highest non‐lethal stress level was related to the cold‐induced shock recorded at 10 °C, where already at the control time activity was significantly reduced. At the hottest suboptimal temperature (30 °C), non‐lethal stress was not immediate, but it increased over time until reaching a critical threshold where the induced stress became lethal. The relevance of measuring non‐lethal stress through the Δ_nl s_ parameter to assess the effect of spray application is further highlighted in the light of research outcomes from El Khoury *et al*.^31^ and Kaya *et al*.^33^ on temperature impact on EPN infectivity. El Khoury *et al*.^31^ studied the control efficacy of seven EPNs over increasing temperatures by assessing the infection of *G. mellonella* larvae; they observed a species‐specific significant reduction in infectivity at higher temperatures. Concurrently, Kaya *et al*.^33^ demonstrated that at suboptimal temperatures, nematodes may enter a quiescent stage, remaining immobile and inactive, and reducing their capacity to infect hosts. Resecco *et al*.,^34^ investigating the *H. bacteriophora* viability as affected by spray application technologies, described a significant decrease of nematode activity along trials duration due to the stress induced by the sprayer components; however, in this case no significant decrease of EPN infectivity was found associated with the reduced EPN activity. Since in this work we have observed an increase of Δ_nl s_ in correspondence to stress augmentation, we can argue that the Δ_nl s_ parameter provides a valuable tool for assessing non‐lethal stress in EPNs, which could have significant implications for infectivity. Future work investigating the correlation between non‐lethal stress parameter and infectivity may be useful in identifying a threshold for maintaining an acceptable efficacy of EPN applications.

Identifying the effects of temperature, exposure time and their interaction on EPN viability (including survival, activity and non‐lethal stress) is crucial to identify the EPNs ‘comfort zone’ to transfer these results to the spray application technology design. Indeed, it is well known that conventional sprayers use a pump to pressurize and propel the spray mixture, as a result its temperature increases due to friction in the hydraulic circuit and continuous pump recirculation.^17^, ^19^ Beltrán‐Martí *et al*.^35^ demonstrated that temperature of the spray mixture increased significantly with the number of passes through hydraulics pumps in laboratory conditions, concretely with 30 passes it increased by 37.6 and 23% for the diaphragm and piston pump, respectively. On the other hand, Fife *et al*.^19^ studied the increment of temperature of 757 L of water recirculation, through hydraulic sprayer under outdoor conditions for an 8‐h workday (9:00 AM to 5:00 PM); temperature of water rose by 5.0 °C, from 15.5 to 20.5 °C, influenced by an average ambient temperature of 23.8 °C. Grella *et al*.^17^ simulating spray application under operative conditions and testing two different sprayers identified that the spray mixture temperature rate increase was linked to the tank dimension and especially to the residual amount of spray mixture in the tank along the spray application duration; anyway the final temperature reached by the spray mixture at the end of the application was in all cases higher than 35 °C when the water used to prepare the spray mixture measured 25 °C (rain water stored in a big tank exposed to the sun during summer time). Referenced studies demonstrated that the spray mixture temperature increases, reaching critical values that may affect the performance of the BCAs. Indeed, temperature not only plays a crucial role directly on EPN viability (%) but also in determining the host infection rate of EPNs.^36^, ^37^ The results obtained in our study are fully in line with previous research showing the impact of temperature on EPN viability.^22^ Indeed, in our study EPN viability was significantly reduced from control (0 min) to 270 min at 30 °C, where about 45–50% of individuals were found alive but inactive, with a potential impact on their final efficacy. On the other hand, at 10 and 20 °C viability remained stable along 270 min exposure time. It has been demonstrated that EPNs are less sensitive to low temperatures than to high temperatures. For example, Kung *et al*.^30^ demonstrated that survival and pathogenicity of *S. carpocapsae* were significantly greater at lower temperatures (in the range 5–25 °C) than at the highest temperature (35 °C). Nilsson and Gripwall^38^ did not observe significant differences on the *S. feltiae* survival after 30 min of recirculation of the EPNs spray mixture at temperatures between 19 and 23 °C. The degree of temperature tolerance varies from species to species and even between strains of the same species^39^ and nematode strains that are used in the biocontrol of pests are active at temperatures between 10 and 30 °C.^40^ This was further in line with our findings that at 40 °C viability drops to 0% (100% mortality) in a few minutes. It is important to note that these findings are based on three widely used EPN species (*H. bacteriophora, S. carpocapsae*, and *S. feltiae*). Other EPN species or strains may exhibit different thermal tolerances or stress responses, and future research should explore the generalizability of these results across a broader range of EPNs.

## 5 CONCLUSION

This research indicates the crucial role of measuring EPN survival, activity and non‐lethal stress to obtain a comprehensive assessment of EPN viability following spray application. Therefore, we recommend the combined use of these parameters in further trials aimed at evaluating in a reliable way the effect of single sprayer components (e.g., pump, filters, pipes, nozzles, etc.) and operating parameters (e.g., hydraulic circuit pressure) on the EPN viability and the stress level (e.g., mechanical, thermal and exposure) induced by each component to the EPNs. The same parameters could be useful to assess the effect of post‐application field conditions, which may introduce additional stressors, such as UV exposure, fluctuating temperatures, and interactions with other environmental factors.

In this context, our findings emphasize the need to assess both survival and activity parameters to obtain an accurate evaluation of EPN viability. While survival provides an indication of the number of live individuals, activity levels are essential to determine the EPNs ability to move and, consequently, their effectiveness in locating and infecting hosts. Additionally, the introduction of the Δ_nl s_ parameter provides a valuable tool to quantify non‐lethal stress, allowing for an early detection of stress effects before they become lethal.

Temperature and exposure time were found to have significant effects on EPN viability, with 20 °C being identified as the optimal temperature to minimize stress. Exposure to higher temperatures (≥30 °C) led to increased stress and mortality, while lower temperatures (10 °C) caused a reduction in activity but did not significantly affect survival. These findings emphasize the importance of maintaining an optimal temperature range during spray application to preserve the viability and efficacy of EPNs.

The study also confirmed that the NaCl stimulation method did not provide a significant advantage in assessing EPN activity, as it failed to reactivate inactive but viable nematodes. Therefore, we recommend using prodding for measuring survival and no stimulation for assessing activity, as they provide the most reliable evaluation of EPN viability.

From a practical perspective, the results emphasize the need for EPN‐friendly sprayer technologies. Future research should focus on validating these findings under field conditions to ensure their applicability to real‐world spray application scenarios. This will help bridge the gap between laboratory studies and practical implementation of EPN‐based biocontrol, informing about how spray systems should be optimized to reduce thermal and mechanical stress on nematodes, ensuring that key components such as pumps, filters, and nozzles do not induce excessive stress that could compromise EPN infectivity. Future research on this topic should (i) assess the effect on EPN survival, activity and non‐lethal stress of exposure to PAE with different technologies under field conditions, and (ii) explore the interaction between non‐lethal stress and infectivity, aiming to establish thresholds for maintaining acceptable levels of EPN efficacy under real‐world conditions. Finally, alternative viability assessment methods and their applicability to different EPN species should be explored to enhance the reliability of EPN viability evaluations.

## AUTHOR CONTRIBUTIONS

Roberto Beltrán‐Martí: Conceptualization, methodology, investigation, data curation, writing–original draft. Marco Resecco: Conceptualization, methodology, investigation, data curation, writing–review and editing. Elena Gonella: Conceptualization, methodology, validation, resources, writing–review and editing, supervision, project administration, funding acquisition. Sofía Victoria Prieto: Validation, investigation, writing–review and editing. Marco Pittarello: Formal analysis, visualization. Cruz Garcerá: writing–review and editing. Patricia Chueca: Writing–review and editing. Alberto Alma: Resources, funding acquisition. Fabrizio Gioelli: Resources, writing–review and editing, funding acquisition. Marco Grella: Conceptualization, methodology, formal analysis, resources, writing–original draft, supervision, project administration, funding acquisition.

## FUNDING INFORMATION

This research was partially funded by the European Union ‐ Next Generation EU, Mission 4 Component 1.5 ‐ ECS00000036 and by the Grant PRE2020‐096256 funded by MICIU/AEI/10.13039/501100011033 and by “ESF Investing in your future”.

## CONFLICT OF INTEREST

The authors declare that they have no known competing financial interests or personal relationships that could have appeared to influence the work reported in this paper.

## Supporting information


**Data S1.** Supporting Information.

## Data Availability

The data that support the findings of this study are available from the corresponding author upon reasonable request.
