# Supplementary material for: Identifying key parameters for reliable assessment of entomopathogenic nematodes viability as affected by spray application stress‐related factors
Source: Pest Manag Sci. 2025 Apr 26;81(8):4799–809. doi: 10.1002/ps.8847 (PMC12268797; doi:10.1002/ps.8847)
Supplement: Supplementary file 1 — Data S1. Supporting Information. [file PS-81-4799-s001.docx]

# SUPPLEMENTARY MATERIAL


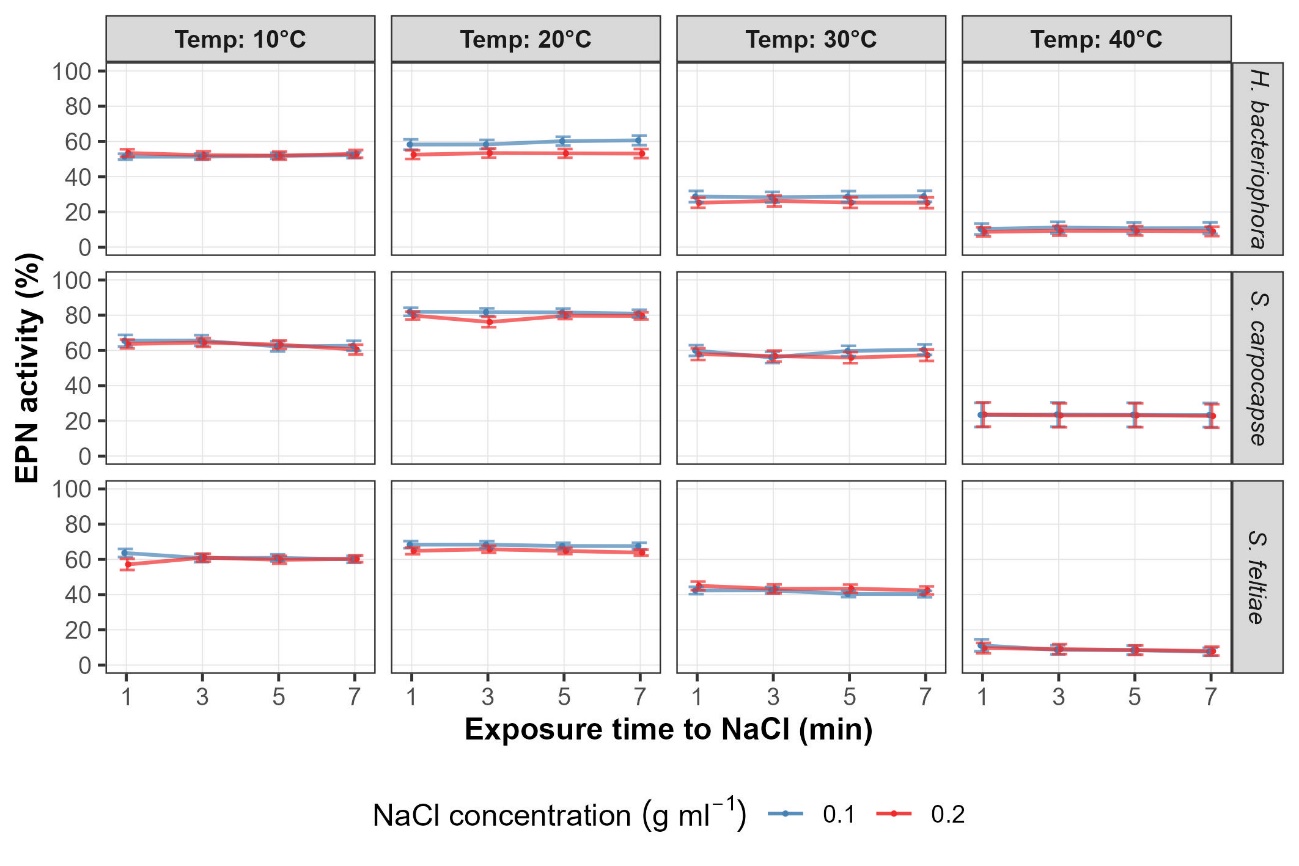


**Figure S1. Relationship between EPN activity (%) and NaCl exposure time (1,3,5 and 7 min) for the two NaCl concentrations (0.1 and 0.2 g mL^-1^) by using chemical stimulation method. Plots are split by EPN species (*H. bacteriophora*, *S. carpocapse* and *S. feltiae*) and temperatures (10, 20, 30 and 40°C) used to induce thermal stress to EPN. Average values ± standard error of the mean (SEM) are represented.**


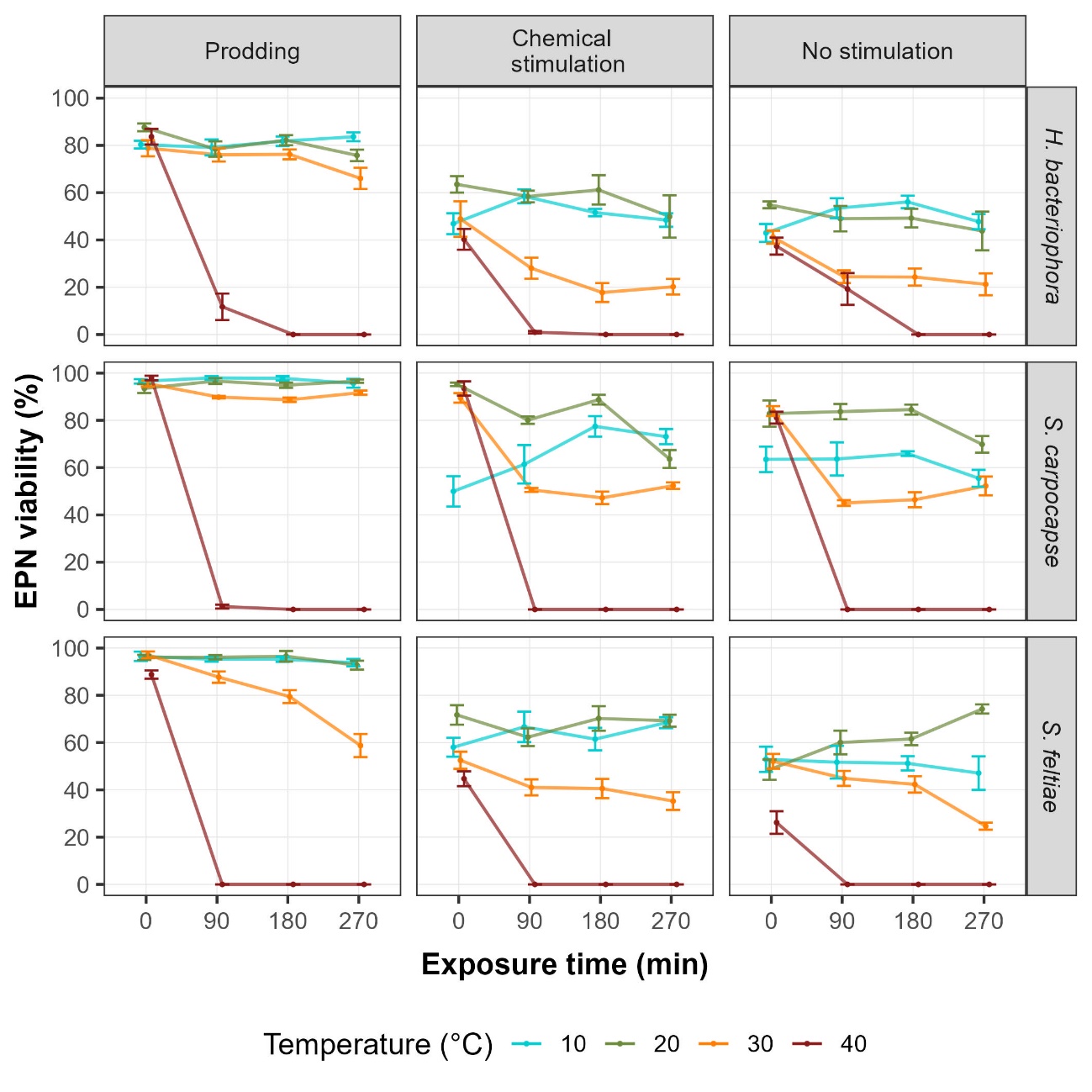


**Figure S2. Relationship between EPN viability (%) and temperature exposure time (0, 90, 180 and 270 min) for the temperatures (10, 20, 30 and 40°C) used as thermal stress to EPN. Plots are split by laboratory measurement method (motility stimulation, chemical motility stimulation and motility not stimulated methods) and EPN species (*H. bacteriophora*, *S. carpocapse* and *S. feltiae*). Average values ± standard error of the mean (SEM) are represented.**

**
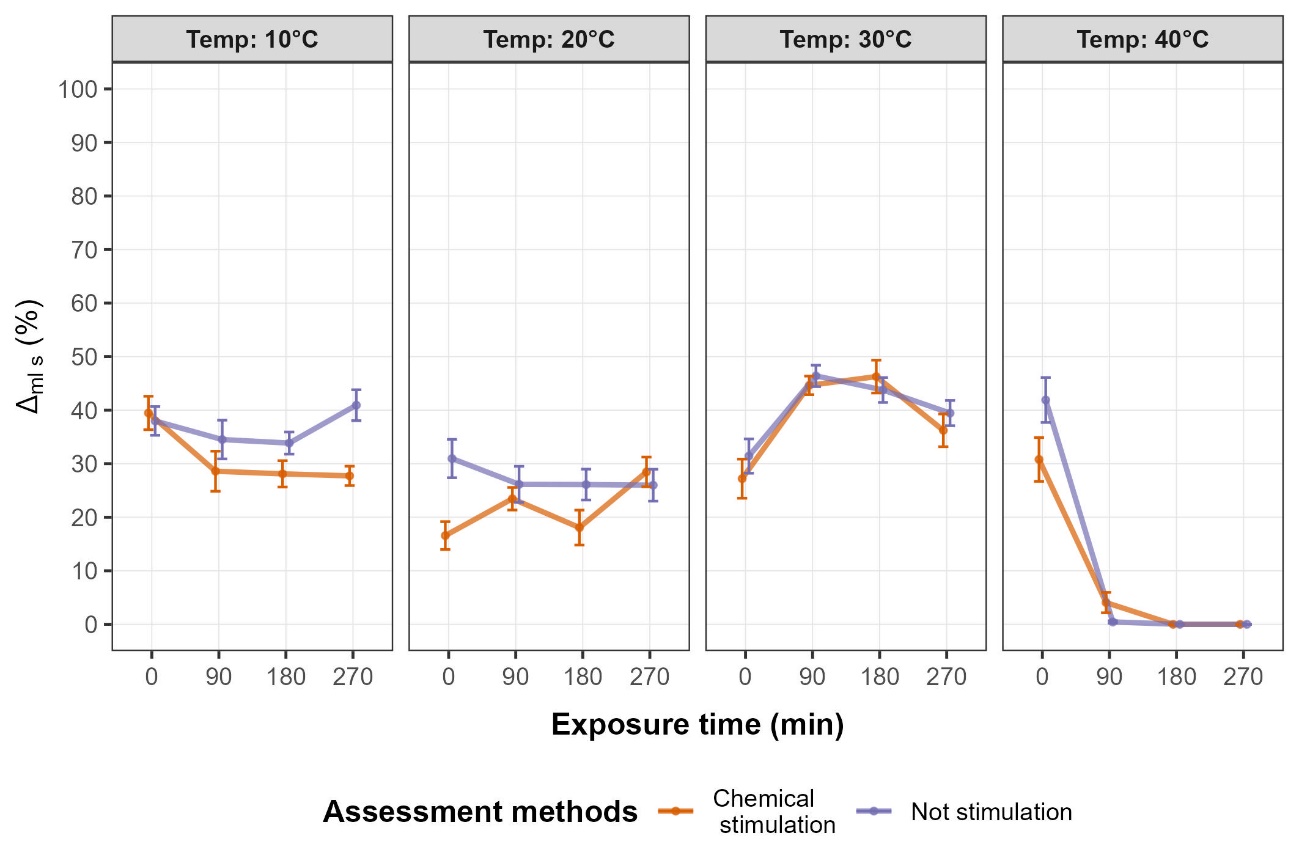
**

**Figure S3. Relationship between Δ_nl s_ (% of live but non-motile nematodes) and temperature exposure time (0, 90, 180 and 270 min) for the chemical motility stimulation and non-stimulated methods. Plots are split by temperatures (10, 20, 30 and 40°C) used as thermal stress to EPN. Average values ± standard error of the mean (SEM) are represented.**
